# Contemporary perspectives on heterotopic ossification

**DOI:** 10.1172/jci.insight.158996

**Published:** 2022-07-22

**Authors:** Charles D. Hwang, Chase A. Pagani, Johanna H. Nunez, Masnsen Cherief, Qizhi Qin, Mario Gomez-Salazar, Balram Kadaikal, Heeseog Kang, Ashish R. Chowdary, Nicole Patel, Aaron W. James, Benjamin Levi

**Affiliations:** 1 Division of Plastic and Reconstructive Surgery, Department of Surgery, Massachusetts General Hospital, Harvard University, Boston, Massachusetts, USA.; 2 Department of Surgery, Center for Organogenesis Research and Trauma, University of Texas Southwestern Medical Center, Dallas, Texas, USA.; 3 Department of Pathology, Johns Hopkins University, Baltimore, Maryland, USA.; 4 Division of Plastic and Reconstructive Surgery, Department of Surgery, University of Michigan, Ann Arbor, Michigan, USA.

## Abstract

Heterotopic ossification (HO) is the formation of ectopic bone that is primarily genetically driven (fibrodysplasia ossificans progressiva [FOP]) or acquired in the setting of trauma (tHO). HO has undergone intense investigation, especially over the last 50 years, as awareness has increased around improving clinical technologies and incidence, such as with ongoing wartime conflicts. Current treatments for tHO and FOP remain prophylactic and include NSAIDs and glucocorticoids, respectively, whereas other proposed therapeutic modalities exhibit prohibitive risk profiles. Contemporary studies have elucidated mechanisms behind tHO and FOP and have described new distinct niches independent of inflammation that regulate ectopic bone formation. These investigations have propagated a paradigm shift in the approach to treatment and management of a historically difficult surgical problem, with ongoing clinical trials and promising new targets.

## Background

Physicians and surgeons have, due to their trades, been long-standing observers of biology. Guy Patin and André Falconet were two such examples in 17th century Paris. As the dean of faculty of medicine of the University of Paris, Patin was an early member of the field of academic medicine; while a frequent target of criticism by his contemporary, the playwright Molière (1), for his rigid profession, Patin penned a great number of informal letters containing his observations on human disease (2). Although obfuscated by scathing condemnations of charlatans, nonmembers of the medical profession, and his study of antimony, his writings also documented fascinating phenomena, including a detailed case (3) involving the progressive ossification of the musculature along a patient’s spine (4). In correspondence with a fellow physician in August 1648, he references Falconet’s writing (5) about how the woman “qui est devenue dure comme du bois,” or “became hard as wood,” perhaps documenting the first known case of ectopic bone formation, or heterotopic ossification (HO).

Similar phenomena, of which there have been multiple independent observations, have since had several names, including myositis ossificans progressiva (6), stone man syndrome (7, 8), and Münchmeyer’s disease (9). These accounts note a predilection for manifesting during childhood or adolescence, with progressive debilitation that begins to affect joints and musculature of the head, neck, and mouth (9) and advancement to fatal involvement and restriction of respiratory structures (10). This disease is now known as fibrodysplasia ossificans progressiva (FOP) (11), with contemporary estimates of prevalence around 0.88 cases per million people (12). Historical documentation of ectopic bone formation is confusing owing to the number of names used to describe these pathologies, with some describing overlapping or identical phenotypes and others conflating distinct diseases.

In 1938, Geschickter and Maseritz published reports of peculiar growths of bone in the setting of recurrent trauma, both in civilian life, such as the condition termed “shoemaker femur,” in which cobblers developed ossifications along their thighs, which were used as a platform for hammering and flattening leathers, and in military personnel, who developed “rifle shoulders” due to the repetitive kickback of their firearms (13). Additional accounts by subsequent German and French physicians, including Riedel (14) and Dejerine (15), further contributed to the collection of cases describing a second variety of ectopic bone formation or HO. These bone formations were described in the context of severe injury to the CNS (14, 15), heralding a growing number of accounts of posttraumatic HO (tHO), involving musculoskeletal injury, including blasts, burns, and deep orthopedic surgeries (16, 17). Unsurprisingly, tHO has been documented in the setting of combat amputations since the Civil War and World War I (18). Indeed, the descriptive literature grew rapidly, especially in the context of contemporary wars, including those in Iraq and Afghanistan (17, 19–21), where complex musculoskeletal polytrauma, such as that resulting from blast and improvised explosive device injuries, increased in incidence (up to 63% of residual limbs; ref. 22), leading to profound tHO presentations. Technological advancements in weaponry with increased blast damage in concert with improved capabilities in evacuation and surgical stabilization have led to more severe antecedent injuries and increased survivability in the setting of historically fatal trauma (18). Improved personal protective gear and prevalence of tourniquet use have increased successful stabilization and presentation to medical centers, where advanced resuscitative techniques have led to increased numbers of survivors and patients with HO (20).

**Figure 1:**
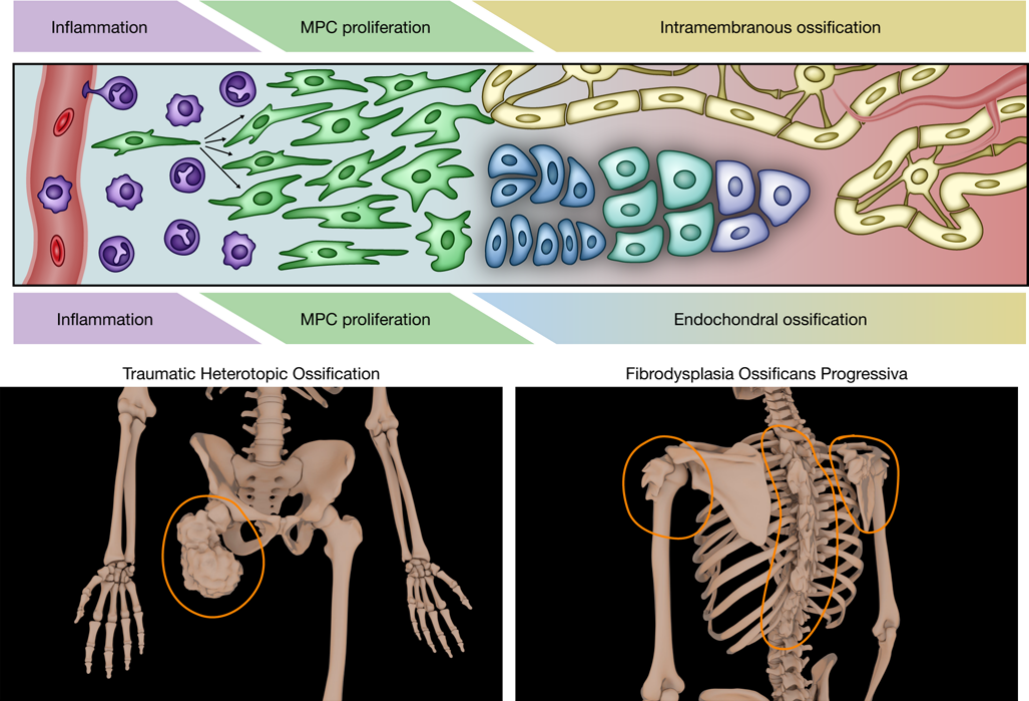
HO induces reactivation of developmental programs found in bone. Bone development in traumatic and genetic forms of HO is initiated by a range of inflammatory responses and recapitulation of developmental bone biology, via both predictable patterns of direct mesenchymal progenitor cell (MPC) differentiation into bone-forming osteoblasts (intramembranous ossification, top progress bar) and the deposition of cartilagenous scaffold via chondroblasts and subsequent infiltration and differentiation of osteoblasts (endochondral ossification, bottom progress bar). These yield robust formation of bony lesions typified in the appendicular skeleton in traumatic HO versus the axial skeleton in genetic forms, i.e., fibrodysplasia ossificans progressiva (FOP) (bottom).

**Figure 2:**
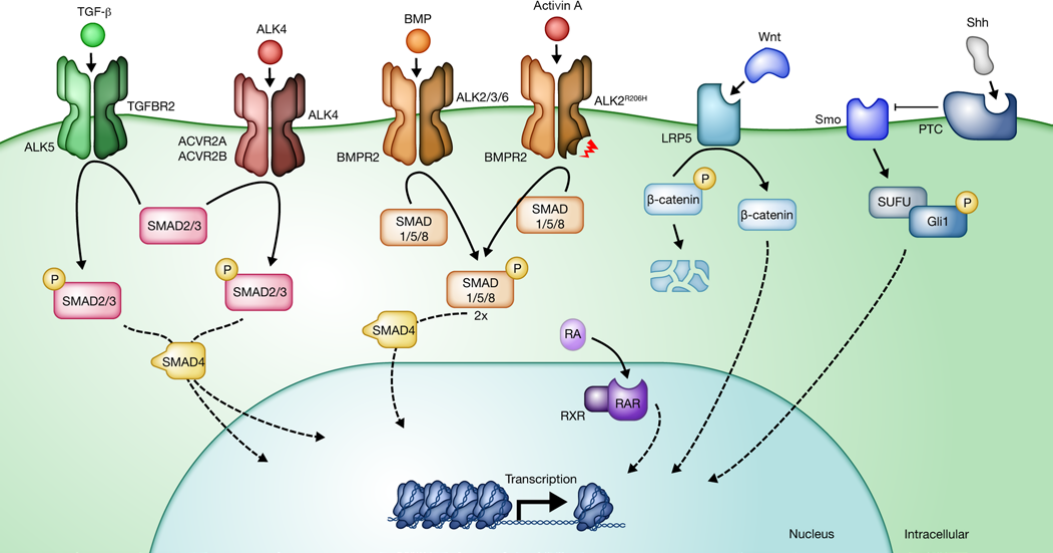
Cell signaling pathways in bone formation. The postinflammatory component of HO formation continues to echo patterns found in normal bone development, including cascades triggered by members of the TGF-β superfamily (TGF-β, activin/inhibins, and BMP) as well as Wnt, Shh, and retinoic acid (RA). These ligand-receptor complexes propagate signaling by cognate secondary messengers including SMAD2/3, SMAD1/5/8, β-catenin, and Smo/Gαs, and RAR-MAPK. These signaling cascades yield transcriptional changes that regulate chondrogenic and osteogenic differentiation.

HO has been well defined in the contemporary literature as a manifestation of reactivation of bone-forming programs that involve inflammatory recruitment; proliferation of local progenitor cells, including chondroblasts and osteoblasts; and remodeling and maturation into mature bone, with specific predilections for musculoskeletal polytrauma sites in tHO or joints of the axial skeleton in FOP (Figure 1). While both tHO and FOP have been demonstrated to reflect aberrant inflammation that triggers endochondral ossification (23–26), the antecedent signals for this convergent programming seem distinct within the existing literature. In both varieties, pathology appears dependent on the behavior of a specific subset of receptors sensitive to TGF-β superfamily ligands, including Alk2 (also known as ACVR1), Alk3 (also known as BMPR1A), Alk4 (also known as ACVR1B), Alk5 (also known as TGFBR1), Alk6 (also known as BMPR1B), and Alk7 (also known as ACVR1C) (25, 27–29) (Table 1 and Figure 2), suggesting possible candidate receptors for medical therapies. However, existing modalities focused on attenuating the inflammatory response, pharmaceutically retarding bony deposition, and poisoning proliferative potential via radiation have yielded limited success, with surgical extirpation typically threatened by recurrent ectopic bone. In these complex settings, many aspects of these diseases remain incompletely understood. Given the divergence in molecular biology, clinical manifestations, and current treatment paradigms, we delineate the contemporary understanding of these pathophysiologies. Despite the rapid characterization and rigorous study of HO, there exists a great need and demand for more robust therapeutics.

## Genetic variants of HO: myositis ossificans progressiva, also known as FOP

### Context.

As described above, nomenclature for the genetically driven form of HO has remained varied. However, descriptions of stone man syndrome seemingly converge to describe the classic progressive worsening of bony lesions, especially across joint spaces, typically in youth or early adulthood, which lead to debilitating loss of joint range of motion and demise. As awareness of and advocacy for the disease known as FOP have continued to increase, diagnosis has also continued to occur earlier and increasingly in pediatric patients, who have been notoriously misdiagnosed historically, leading to harmful biopsies and erroneous treatments. This growing body of knowledge also exhibits a curious diagnostic exam finding: congenitally shortened great toe with missing or abnormal first phalanx and metatarsal, commonly with valgus deformity.

### Natural disease progression.

FOP is a consequence of a sporadic gain-of-function mutation in the ACVR1 receptor that leads to induction of a major pro-osteogenic signaling pathway. Proper management primarily relies on early and accurate diagnosis, as treatment is primarily prophylactic, including precautions to minimize physical traumas. Historically, patients would describe episodes of severe swelling or painful lesions (30); these flares were often erroneously diagnosed as oncological tumors. Such incorrect diagnoses were especially problematic, as they indicated biopsies and introduction of iatrogenic traumas that would further propagate or incite HO formation. Furthermore, events that are typically benign for most patients, including intramuscular immunizations, dental work, minor bumps, or even viral illnesses, can trigger flares and HO formation (30, 31). In patients with FOP, HO formation typically starts in dorsoaxial regions and slowly progress outward. These flares are chronically cumulative (32), confining a majority of patients to a wheelchair by the third decade of life and leading to critical cardiorespiratory failure secondary to rigid fixation of the chest wall and thoracic insufficiency (30).

### Treatments.

For FOP, primary treatment still remains preventative — for example, proactive dental care to limit the need for dental procedures that require mandible injections, avoidance of intramuscular immunizations, prevention of falls, and pulmonary therapy to maximize function and reserve (30). The principal management for HO remains surgical excision, which is often complicated by recurrence, which is nearly universal in FOP (30, 33). Given these constraints, genetically driven HO has been largely restricted to more conservative, supportive measures to mitigate flares and retard HO lesion progression via the use of steroids and NSAIDs, with varied results (34). Indeed, data-driven therapies for these lesions have remained sparse. Some groups have reported the use of antiinflammatory modalities, including mast cell and leukotriene inhibitors to target the inflammatory components of early FOP lesions along with bisphosphonates for more refractory flares (35, 36); however, the effects are modest, with unclear protocols for proper timing of therapies. Unfortunately, there is no proven therapy to alter the natural history of the disease (37). However, promising therapies, including palovarotene (RARγ agonists; refs. 38–40), activin A antibodies (41, 42), saracatinib (ALK2 inhibitors; ref. 43), and rapamycin (mTOR inhibitor; ref. 44), are currently undergoing phase II or III clinical trials that may yield directed therapies for patients with FOP.

### The TGF-β superfamily.

Both FOP and tHO have been canonically described as involving signaling cascades within the TGF-β superfamily. Two of the three members of the TGF-β ligand superfamily, TGF-β1 and bone morphogenetic protein (BMP), share a signaling motif that is curiously indirect, as each specifically binds its type II receptor (45, 46) — TGFBR2 (47) and BMPR2 (48), respectively — which, in turn, phosphorylates a nearby type I receptor (Alk5 and Alk2, -3, and -6, respectively; ref. 49). The activated receptors phosphorylate serine residues within the conserved SSxS motif at the carboxy terminus of R-SMADs (SMAD2/3 for TGF-β and activin, SMAD1/5/8 for BMP). The activated R-SMADs form trimeric complexes with a co-SMAD (SMAD4) and translocate to the nucleus to modulate target gene expression (50–52). TGF-β1, the most abundant and ubiquitously expressed isoform (53), has been extensively characterized owing to its role in proliferation and differentiation of cartilage and bone formation (54). Similarly, BMPs have also been extensively characterized in bone development and verified as causal signals capable of inducing pathological excess bony tissue when overexpressed (48). Indeed, hyperactive ACVR1 signaling in FOP macrophages abnormally increases secretion of TGF-β and proinflammatory cytokines (55). Elevation of active TGF-β recruits mesenchymal progenitors to the HO site, while administration of TGF-β–neutralizing antibody effectively attenuates HO progression in both FOP and acquired HO models (29). In addition to canonical SMAD pathways, TGF-β/BMP family ligands also transmit signals via non-SMAD cascades, including MAPKs, small GTPases, PI3K/AKT/mTOR, and TGF-β–activated kinase 1 (TAK1) (56). Notably, FOP macrophages exhibit prolonged NF-κB and p38 MAPK activation without significant changes in SMAD1/5 phosphorylation, indicating dysregulated TAK1 activation in HO (55). Furthermore, TAK1 has also been implicated as a regulator of mesenchymal stem cell (MSC) proliferation via stabilization of YAP/TAZ (57). Thus, SMAD-independent TAK1/MAP3K7-dependent propagation of TGF-β via activation of upstream TAK1-binding proteins (58–60) is under active investigation as a candidate target.

### The FOP mechanism.

It has become clear that FOP, while involving signaling pathways that overlap with tHO, is physiologically distinct. Another member of the TGF-β superfamily, activins, has been characterized in a predominantly endocrine context; it was originally discovered as a gonadal protein that stimulates release of follicle-stimulating hormone (61). Because of interactions with follistatin, it was previously inferred that activins A and B bind their type II receptors (ActRII or ActRIIB) and subsequently phosphorylate their corresponding type I receptors, ALK4 and ALK7, respectively. Moreover, activin A contributes to an inhibitory tone on BMP-Alk2–mediated signaling upon binding of ACVR2A/B (62) with activation of SMAD3-mediated inhibition and via the formation of nonsignaling complexes (41). Aberrant behavior of ACVR1 has been shown to be a central locus for FOP pathogenesis (27, 63), as expression of human mutant ACVR1 (ACVR1^R206H^) in mice results in FOP-like disease (27, 63). This single arginine-to-histidine mutation in *ACVR1* leads to a paradoxical activation of downstream signal upon activin A binding of the type II and type I receptor complex, increasing the same intracellular signals typically seen upon binding of SMAD1/5/8 by BMP (28). Notably, contemporary work has suggested a slight modification to the conceptualized mechanisms of signal propagation. A recent optogenetics study revealed that hyperactive ACVR1^R206H^-mediated SMAD1/5/8 activation occurs via activin A–dependent receptor clustering and that the role of type II receptors ACVR2A/B is only structural and does not require upstream kinase activity, as is typical of other TGF-β superfamily ligands (64). The kinase domains of type I receptor ALK2 and type II receptor BMPR2 form a heterodimeric complex that serves as a scaffold for subsequent assembly of active tetrameric receptor complexes, thereby enabling SMAD activation (65). Nevertheless, this net increase in pro-osteogenic signaling has been well characterized as the antecedent event in genetically driven HO formation. Importantly, investigators have recently validated that activin A antibody robustly attenuates FOP-associated HO in animal models (28).

### Molecular targets.

While the ACVR1 gain-of-function mechanism of FOP HO formation remains largely independent of a robust antecedent inflammatory response (a small putative role of mast cells and macrophages has been observed; ref. 66), the signaling pathway is heavily contingent on activin A levels. Circulating levels of activin A have previously been shown to increase following inflammatory insult (67), which may explain the clinical challenge with FOP, where more subtle inflammation, even without antecedent trauma, can incite soft tissue swellings or flare-ups (68) once aberrant activity of a receptor has been induced. Thus, the activin A/ALK2/follistatin axis has undergone intense scrutiny with several ongoing phase II clinical trials strategically targeting several of these factors for the mitigation of HO in FOP. Two additional targets worthy of mention are the Hif1α/mTOR axis, downstream signaling cascades integrating hypoxic and anabolic signals important in bone formation (69), and endogenous retinoid signaling, whose inhibition permits chondrogenic differentiation (70, 71). Rapamycin has been shown to profoundly mitigate bone formation in FOP models following antecedent injuries downstream of activin A activity and attenuates mesenchymal progenitor proliferation and HO anlagen in animal models (72) and in two human case reports (73). A formal trial centered at Kyoto University has completed enrollment in phase II studies. Historical data have also demonstrated efficacy of RARγ agonists in ligand-independent mouse models with FOP-like lesions (38) as well as ACVR1^R206H^ mice (38, 39, 74). Phase II and III clinical trials investigating these therapeutics have also been ongoing (Table 2). The phase III MOVE trial for palovarotene has most recently reported “a mean annualized new HO volume reduction of 62% when compared with nontreated participants” and is still undergoing active investigation (75). Notably, the LUMINA-1 trial of garetosmab (anti–activin A, Regeneron) and the MOVE trial of palovarotene (RARγ agonist, Ipsen) have both undergone partial holds due to investigation of serious or fatal adverse effects and premature growth plate fusions, but remain ongoing with subsequent protocol adjustments, including restriction of dosing to patients 14 years or older in the MOVE trial in early 2020. LUMINA-1 has scheduled trial resumption with progression to phase III beginning in early 2022. Several Alk2/ACVR1 inhibitors have emerged as possible candidates, although trials are still in early phase I (DS-6016a [Daiichi Sankyo], INCB000928 [Incyte], and IPN60130/BLU782 [Ipsen]).

## Nongenetic tHO

### Inflammatory priming for ectopic bone.

In the context of musculoskeletal polytrauma, no genetic mutation drives the formation of ectopic bone. Instead, tHO is characterized by a critical threshold of injury that induces inflammation and hypermetabolism that precipitously dysregulates normal tissue repair (76, 77). HO manifestation is conserved across all tissues, with the initial response following injury characterized by an influx of neutrophils (78) and monocytes (79, 80) that propagate innate immunity via NETosis and TLRs (81), clearance of debris (82), and antigen presentation (83) to govern crosstalk with the B and T cells of the adaptive system (77, 84, 85). Mast cells are also described in ectopic bone (85, 86). Subsequently, in the late inflammatory to early proliferative phase, a large population of myeloid cells, composed of a spectrum of macrophages, populate the inflammatory milieu and serve as highly secretory entities (87, 88) that exert autocrine and paracrine effects (via CCL2, TNF-α, CXCL1, CXCL2, IL-3, IL-6, IL-10, MCP-1, and TGF-β) (89, 90) on nearby mesenchymal progenitor cells, which in turn aberrantly differentiate into hard connective tissues (91). In the context of neurogenic HO, macrophage-derived oncostatin M contributes to HO in mouse and human tissues (88). As macrophages within the newly forming HO anlagen begin to polarize toward an antiinflammatory phenotype (80, 92), additional inductive signals like TGF-β1 (54) are critically upregulated at the ectopic bone site (29, 80, 90), and reduction of these signals impairs the HO phenotype (29, 90).

### The TGF-β superfamily revisited.

While inhibition of SMAD-independent signaling via TAK1 can attenuate HO formation in acquired HO (93), these pathways have not been validated yet in humans. Furthermore, anti–activin A is a very promising therapeutic for FOP; however, we have previously demonstrated that the compound is ineffective for treatment of tHO, reinforcing the divergence in mechanisms of FOP and tHO. Notably, the influence of activin A in extremity HO remains an area of interest. For example, Pacifici and colleagues demonstrated effective attenuation of subcutaneous HO with anti–activin A (94). Both of the HO mouse models used express WT ACVR1 and showed increased activin A production upon HO induction. Interestingly, single-cell RNA sequencing (scRNA-Seq) data displayed that activin A (encoded by *Inhba*) was mainly expressed in smooth muscle cells and pericytes in an induced tHO model, whereas it was coexpressed with *Sox9* in recruited progenitor cells in the BMP2-implant HO model (94, 95) (Tables 1 and 3). Therefore, the activin A expression pattern partially explains the discrepancy between the tHO model and the BMP-implant HO model in response to anti-ACVR1 for HO reduction, warranting further studies. Nonetheless, studies that have focused especially on the inflammatory phase have shown that direct depletion of macrophages markedly reduces tHO formation across exogenous BMP, spinal cord injury (SCI), and burn/tenotomy polytrauma HO models (84, 87, 90). Notably, HO of the temporomandibular joint (TMJ) has become an area of increased interest due to its clinical implications, in particular its intersection with FOP HO (96), including trismus and restricted mouth opening (96, 97).

### TMJ HO.

HO of the TMJ is defined as presence of extraskeletal bone around the TMJ. TMJ immobility, or ankylosis, can subsequently result in malnourishment, pain, and an overall decrease in the quality of life. A genetic component has been reported in connection with FOP (96), as trismus has been observed in multiple case reports (96, 98). FOP-associated HO formation in the maxillofacial region tends to recur after surgical excision; thus patient quality of life is an important factor in deciding when to surgically intervene (99). FOP-associated complications can arise during intubation as a result of TMJ ankylosis and spinal rigidity (100). The TMJ is a highly complex joint composed of the mandibular condyle; the articular capsule, an articular disc between the condyle and the glenoid fossa; the synovium; the temporal articular fossa or the glenoid fossa; and articular ligaments (101). As a result of the location and local microenvironment, the TMJ is also in a precarious position for tHO development (102). The cellular mechanisms of TMJ HO seem to echo motifs found in extremity HO. Xiao et al. collected cells from ankylosed joint specimens that were shown to have MSC-like properties (103). In classic osteogenic media, induction of the MSC population results in conversion into osteogenic cells, as demonstrated with alizarin red and alkaline phosphate assays. Aberrant induction of MSCs results in the upregulation of the BMP cascade and consequently TMJ HO (97). Targeted radiation was shown to reduce TMJ HO recurrence by 50%, with xerostomia as the only attributable side effect (104). Other anti-HO modalities like indomethacin have had little clinical documentation, with only one case report in which indomethacin prevented HO recurrence following resection in the setting of TMJ ankylosis (105). Moreover, bisphosphonates may have untoward consequences, including pro-ankylotic effects (106) and known risk of jaw osteonecrosis. While theoretically sound, such studies have faced further contraindications due to the relative frequency with which they are skewed toward pediatric patients.

## Contemporary clinical management of FOP and tHO

Based on current understanding of the disease process, existing treatment paradigms primarily target mitigation of the inflammatory burst. Current treatment strategies for patients with preexisting HO formation who present clinically with pain and decreased range of motion are relatively limited; they comprise physical therapy and/or surgery following extensive maturation of bone (at least 6 months to a year after injury). Surgical HO excision improves range of motion and restores limb functionality (107); however, complete resection of HO is difficult, given its vascularity, and is complicated by soft tissue deficits secondary to wound scarring, loss of domain, contractures, and pain.

### Diagnostics.

Diagnostic modalities contribute to the multifactorial clinical challenges with HO. While plain radiographs and CT scans can detect mature HO and bone, they perform poorly for detection of early or potential lesions (108–111). Furthermore, for pediatric diseases like FOP, exposure to large amounts of radiation is typically contraindicated. Diagnostics that provide more rapid visualizations like ultrasound and spectral ultrasound imaging have been proposed but are limited by operator variability and specificity (112, 113). MRI can detect increased vascularization and density in the acute phase but faces pragmatic and logistical limitations in becoming a standard diagnostic modality (114, 115). Bone scintigraphy, FDG-PET, single-photon emission CT, Raman spectroscopy, and noninvasive infrared spectroscopy constitute a spectrum of possible meritorious modalities but without robust validation in human contexts (16, 111, 116, 117). Limitations of these approaches include inability to distinguish new bone formation and marked operator dependence (118), limited additional information in comparison with CT, difficulty in distinguishing differential diagnoses like malignancy or infection (111), possible false positives, including detection of simple inflammation obscuring detection of clinically relevant ectopic bone, and high operational costs (118–120). Especially in FOP, clinical diagnoses are typically sufficient based on history, including classic deviations in the great toes and the presence of rapidly developing soft tissue lesions. Owing to imaging modality limitations, investigators have also demonstrated the possible predictive value of serum biomarkers, including proteomic profiles of patients developing HO that have implicated known osteogenic signals like osteomodulin, osteocalcin, and collagen (121), and inflammatory cascades, including IL-6, IL-10, and MCP-1 (73). Interestingly, in the context of neural injury, decreased levels of α_2_-HS glycoprotein and increased calcium, D-dimer, BMP, and CRP were found to correlate with neurogenic HO formation (122). In addition to these classic proxy laboratory values for inflammation, cell-free nucleosomes, as fragments of neutrophil extracellular traps (NETs), also correlate with disease outcome measures (specifically in community-acquired pneumonia; ref. 123). NETs have also been shown to critically regulate inflammatory influx and downstream HO (124). Biomarkers appear promising as a supplemental method for the detection and prediction of HO but require further validation.

### Treatment of tHO and FOP.

HO presents a challenging surgical problem. Extirpation would be the indicated remedy; however, varying difficulties in access based on location (extremities vs. spine/ribs) and the high risk and threat of recurrence, often worse in secondary presentation, have largely been thought to be significant contraindications to aggressive surgical solutions. In posttraumatic settings, even in the presence of prohibitive comorbidities including nerve entrapment, pain, resorption of underlying normal bone with pathological fractures, ulceration, and wound formation are managed supportively while awaiting a washout period of 12 to 24 months (22, 107). These paradigms have recently been subjected to increasing scrutiny, especially due to the significant functional impairments downstream to large HO lesions. Surgical resection remains controversial; however, in cases in which HO forms specifically around joint spaces (hip, elbow, knee), earlier resection with early mobilization has been successfully documented (107, 125–127). Resection is performed conservatively, with ectopic bone removed in small wedges with meticulous hemostasis given the typical increased vascularity in HO lesions (128). Nevertheless, prevention and medical prophylaxis for the acute postinjury period remain sparse.

As treatment options for HO after diagnosis are limited, prophylactic measures in high-risk populations are commonly utilized. NSAIDs, via purported mechanisms of prostaglandin inhibition (129) and direct suppression of osteoblast cell cycle progression (130), have remained the limited gold standard. In an early account, Ritter and Sieber retrospectively evaluated a cohort of patients with hip arthroplasty who were operated on before and after initiation of routine indomethacin treatment for high-risk individuals. Those who received indomethacin (25 mg three times daily for 6 weeks) had an absolute risk reduction (ARR) of 4% in Hamblen grade 2–3 formation (one-third to complete involvement of the hip space). Moreover, all HO formation after program initiation occurred in those overlooked for indomethacin treatment (129). Subsequent studies have corroborated these observations across a range of doses: 150 mg daily (either 50 mg three times per day or 75 mg two times per day) for 3 to 6 weeks resulted in 28% ARR of late HO in SCI (131) and complete prevention of Brooker grade 2–4 HO in total hip arthroplasty (132). Validation in other settings, including burns (133) and combat, has been fairly limited, as acute trauma states necessitate prioritizing of life-saving procedures, sometimes at the expense of future morbidity (20). Other NSAIDs, including 200 mg celecoxib twice daily for 3 weeks, have also reduced HO formation (10% ARR in cohorts of hip arthroscopy; ref. 134) by targeting prostaglandin-mediated inflammatory cascades, although optimal dosing has yet to be confirmed (133, 135). Effectiveness of HO prophylaxis is notably varied among orthopedic procedures (136, 137). Radiation therapy significantly reduces HO incidence compared with indomethacin and is currently the preferred prophylaxis among orthopedic surgeons (138), though some studies find no difference between indomethacin and radiation for HO prevention (139). Radiation doses ranging from 10 to 20 Gy fractionated over 5–10 doses (2 Gy/treatment) have shown significant attenuation of heterotopic bone with ARR of 55% or more when introduced within 48 hours of surgery (140). However, NSAIDs, radiation, and less favored treatments such as corticosteroids (31) and bisphosphonates (141) also have prohibitive side effects, especially in pediatric populations, including GI, renal, and cardiovascular effects, impaired bone/wound healing issues, electrolyte disturbances, jaw osteonecrosis, transient effects, and theoretical oncological risk (142–148). While surgical extirpation is dogmatically more tenable in posttraumatic contexts compared with FOP, once the antecedent trauma is healed, surgery inherently incites additional trauma that may promote recurrence, especially in patients with neurological etiology (125, 126). Thus, tHO has faced limitations, with available treatments awaiting further investigation. FOP faces similar limitations in therapeutic options; however, flares are unique to FOP pathophysiology, and their management must be considered. The most recent International FOP Association guidelines recommend steroid prophylaxis (2 mg/kg/d of prednisone, up to 100 mg, for no more than 4 days; or a high dose, i.e., 20–30 mg/kg, of prednisolone i.v. for alternating days in a hospital setting) for significant blunt muscular trauma, necessary dental/surgical procedures, and any emergent flares, especially of the limbs and jaw (37). Unlike for acquired HO, there are no established studies or evidence demonstrating clinical, preventative benefit for FOP. Outside of symptomatic management for pain and inflammation with NSAIDs during flares or arthropathy, the range of proposed therapeutics, including bisphosphonates, chemotherapy, and other miscellaneous agents, remain ineffective, similar to observations in tHO.

## Contemporary science, investigative models, and future directions for tHO

### Animal models.

Multiple animal models have been used to study acquired HO, including implantation, hip arthroplasty, immobilization/manipulation, Achilles tenotomy, trauma introduction, and irritant/material injection (149). The BMP implantation model is one of the most common and involves injection of BMP2A, Matrigel with BMP, or BMP2/4-overexpressing cells into muscle bellies, resulting in endochondral ossification (150–152). Additional models approximate neurological injury, including SCI (153). While recapitulating the HO phenotype, implanted materials introduce foreign bodies aberrant to normal physiology or the pathophysiology of HO. Furthermore, injections of local irritants like ethanol or hydrochloric alcohol produce unreliable phenotypes (154). More clinically translatable models include hip arthroplasty, which has been validated in rabbits (155); rat extremity blast injury (156); simulated combat trauma by shockwave in sheep (157); and Achilles tenotomy with or without (158) concomitant burn (a reproducible, controlled polytrauma model) (159).

### Tools for genetic interrogation and isolation of mesenchymal progenitors.

Transgenic mice have facilitated development of a variety of lineage tracing models. The use of several Cre drivers has provided incredible insights into the etiology of cells that contribute to HO formation. Induction of colorful Cre-linked reporters, such as endogenous fluorophores (GFP, RFP, tdTomato, or mT/mG), allows subsequent histological analyses to highlight the cell types found within the local environment. Several studies have identified progenitor cells in HO, using Cre drivers, including *Prx-Cre* (160–162) from paraxial mesoderm, *Scx-Cre* (63, 163, 164) from (peri)tendonous structures, *Tie2-Cre* (151, 165, 166) from local pericytes, *Gli1-Cre* from osteoblast precursors (167–169), *Glast*-*Cre* from nonspecific neural and nonneuron-derived progenitor cells (170, 171), and *Hox11a-Cre* (172, 173), which labels all hind-limb skeletal lineages and progenitors of HO. A more expansive list of HO lineage tracing studies can be found in Cholok et al. (146).

### Benchside tools for investigation of mechanisms and candidate targets.

Several scientific techniques both old and new have been integral to expanding our understanding of the pathophysiology of HO. Examples include high-resolution micro-CT (174) for in vivo imaging and segmentable 3D volumes (175), confocal microscopy for high-resolution imaging with capability to produce 3D *Z*-stacks and reporter/fluorophore multiplexing (176, 177), Western blotting, and flow cytometry for cell identification and sorting (178). Analysis of HO-associated transcriptional activity has been revolutionized by the advent of next-generation sequencing (NGS) technology and bioinformatics methods. NGS facilitates high-speed, multiplex sequencing of tissue samples with cellular resolution throughout the progression of HO formation. Assay for transposase-accessible chromatin using sequencing (ATAC-seq) has been used to determine the epigenetic changes that occur during HO progression and highlights accessible regions of chromatin to infer genes likely to be transcribed (179, 180). ATAC-seq has also been performed at a single-cell level (scATAC; ref. 181) to find changes within specific cell populations following injury. Similarly, RNA-Seq has been employed either on whole-tissue digestions of HO anlagen or within single-cell harvests from the injury site, allowing for high-throughput detection of novel, differentially expressed genes and pathways that may serve as effective pharmaceutical targets (182, 183). Centralization of NGS data sets (see Table 4 for HO-associated GEO database entries) has only accelerated discovery. NGS technology has been expanded to include spatial transcriptomics, which allows visualization of where RNA is being transcribed on a histology section, although the technology does not yet allow for single-cell spatial resolution (184). Combined platforms also allow for gathering of both epigenetic and transcription information from the same cell as well as transcript and protein information (CITE-Seq/mass spectrometry) (185).

### Human models.

Outside of clinical drug trials, contemporary implementation of human models has remained relatively limited. Fairly recent generation of induced pluripotent human stem cells has been described from cultivation of cells collected from patients with FOP (186) or discarded primary teeth (stem cells from human exfoliated deciduous teeth [SHED]; ref. 187), and through introduction of FOP mutations through gene editing technologies including CRISPR/Cas9 (188), with phenotypic validation of increased endochondral ossification phenotypes in vitro (189). Notably, these technologies are being implemented into drug discovery/validation pipelines, as seen with rapamycin (190) and saracatinib (191). Other similar models incorporating primary tissues include excision of neurogenic HO and surrounding muscle with sequencing data uploaded to public repositories (88), and primary connective tissue cells harvested from patients with ossification of the posterior longitudinal ligament that subsequently are exposed to cyclical mechanical stresses to approximate HO (192). With the aforementioned technological advances, increasingly granular delineation of the human pathophysiology of FOP will be possible and facilitate translation and validation from bench to bedside.

### Extrainflammatory pathways.

**Figure 3:**
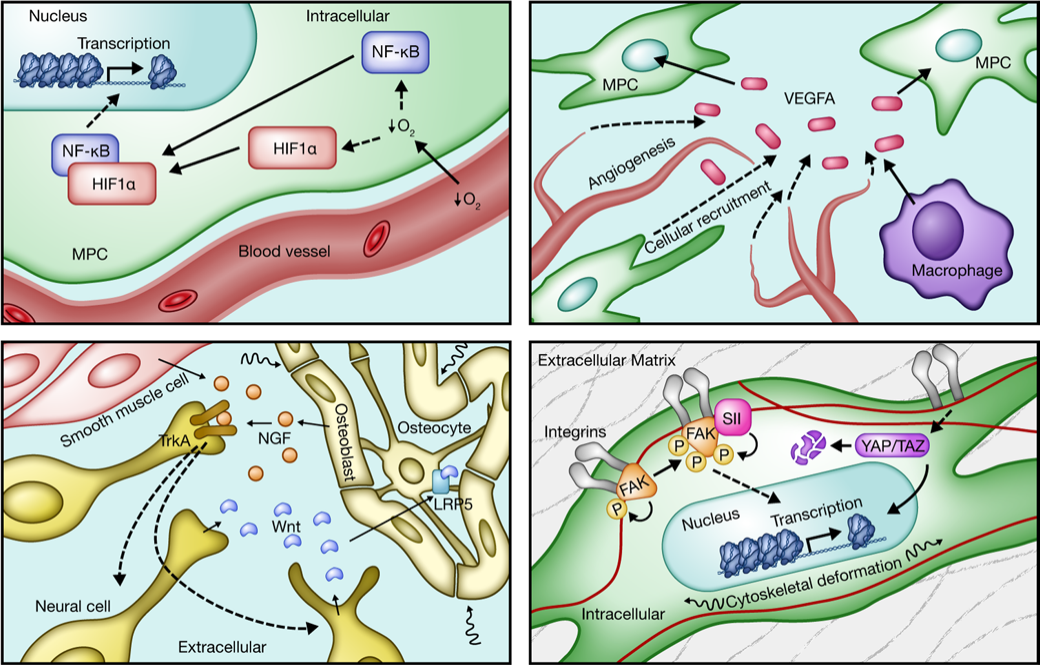
Extrainflammatory pathways that regulate HO formation. Contemporary work has extensively expanded the understanding of regulatory effects on the HO program. Extending from existing work investigating developmental bone biology and fracture healing physiology, several independent niches have been found to impact the formation of ectopic bone, particularly in the setting of posttraumatic HO. Representative topics include vascular and hypoxia signaling pathways involving VEGFA/VEGFR1, Hif1α, endothelium, and perivascular cells (top left and top right); nerve and perineural structures along with associated neurotrophic factors, e.g., NGF and receptor TrkA (bottom left); and the effect of mechanical deformation and forces exerted on progenitor cells residing/migrating through stromal substrates that yield downstream activation through interaction of integrins and FAK, YAP, and TAZ (bottom right).

Many discoveries regarding HO formation through intramembranous and endochondral ossification have extensively borrowed from the developmental biology of bone formation and fracture healing (26). Given the parallels with the role of inflammation in connective tissue formation, especially with respect to macrophages and their influence on neighboring progenitors (90, 91, 193), there has been increased appreciation of regulatory forces that underlie the paradigm of dysregulated inflammation, including upstream or parallel programs involving vascular differentiation and hypoxia signaling via Hif1α and VEGFA (72, 162, 194–197), mechanotransduction and extracellular matrix organization (180, 198–201), and neurotrophic/neuroinflammatory factors and supportive niches, such as NGF, calcitonin gene–related peptide (CGRP), and substance P (SP) (86, 87, 202–209) (Table 5 and Figure 3). With several of these new niches, additional technologies have been incorporated into the study of mesenchymal progenitor cell behavior, including the fabrication of fibrous matrices with electrospun dextran methacrylate (180, 210). The increasing tunability of experimental substrates has further facilitated the increased granularity in cellular data for both animal and human cell investigations.

### Neural regulation of HO.

The influence of neural signaling on ectopic bone formation has become an emerging area of intense focus due to observed correlations in wounded veterans and civilian populations and associated medical challenges (16, 144, 211–213), highlighting the relationship between aberrant bone formation and nerve pathways. The periosteal bone surface is covered by primary sensory and sympathetic axons (214). Sensory nerves are key regulators of bone formation and regeneration after injury (208, 215). Previous studies have demonstrated that surgical and chemical denervation of sensory nerves in murine models reduces bone formation and impairs fracture healing (216, 217). Similar outcomes were observed in patients, where nerve dysfunction delayed skeletal repair (218), making it clear that nerve signaling directly regulates bone repair. In the context of HO, coregulation of nerves and bone has been observed, providing further evidence of this relationship. Moreover, HO has been frequently observed in patients with paroxysmal sympathetic hyperactivity (219, 220), linking HO to the peripheral nervous system. The peripheral nervous system contributes to HO through neuroinflammation, potentially via release of different molecules such as SP and CGRP (87, 203, 205). In addition, BMP2 release promotes neuroinflammation and HO (221). Using a mouse HO model, Salisbury et al. demonstrated that activated sensory nerves participate in HO development and inhibition of nerve activation significantly reduces HO (86). Moreover, HO induction recruits mast cells to the nerve and promotes bone formation (66). Altogether, these findings indicate that HO induction depends on neural inputs. NGF is essential in the development and maintenance of neurons in the nervous system, while the high-affinity NGF tropomyosin receptor kinase A (TrkA) is densely present on innervated bone surfaces. Studies by our group have elucidated how NGF/TrkA signaling plays an essential role in calvarial bone healing and stress fracture repair (208, 222), implicating skeletal sensory nerves as an important mediator of bone formation. Further, in an extremity injury model, NGF-mediated axon innervation accompanied tHO (209). In our study with this model, surgical denervation impaired axonal ingrowth and delayed cartilage and bone formation. Likewise, either NGF deletion or TrkA inhibition delayed axonal invasion and heterotopic bone formation. Thus, the developing narrative on regulation at the neural level of in vivo posttraumatic programs indicates further investigation of peripheral axon-derived messengers and potential mechanisms of additional intertissue crosstalk, including bone nerve, nerve inflammation, and nerve vasculature (Table 5).

### Future modalities for FOP and tHO.

Given the discussion of contemporary perspectives on and understanding of FOP and tHO, new targets for effective and specific therapeutics have begun to rapidly expand. Indeed, for FOP, several clinical trials are already under way (Table 2). For tHO, there is an expanding literature delineating the axes of regulation for both recruited inflammation and bone maturation, highlighting attractive candidates for further study. Understandably, previous theoretical candidate therapeutics focused directly on interrupting progression through chondrogenesis and osteogenesis. The discovery of effective attenuation of bone and the putative tunability of transcriptional programs at the progenitor level through modulation of extraosteogenic and inflammatory axes highlight a paradigm shift in the targets and treatment strategies for ectopic bone formation. Several antibody therapeutics developed for oncology and degenerative diseases that may be effective for treating HO are already FDA approved (bevacizumab, anti-VEGFA; ref. 223) or are in the final stages of FDA approval (tanezumab, anti-NGF; refs. 224, 225). Additionally, insights into the efficacy of rapamycin in FOP may also extend to tHO (226). There is a growing body of literature, cutting-edge investigational methods and techniques, and ongoing clinical trials that provides hope that mitigation of these debilitating diseases will be an imminent reality. Such a future is the direct consequence of the incredible collaborative efforts of tireless scientific investigators, advocates, clinicians, patients, and families.

## Figures and Tables

**Table 5:**
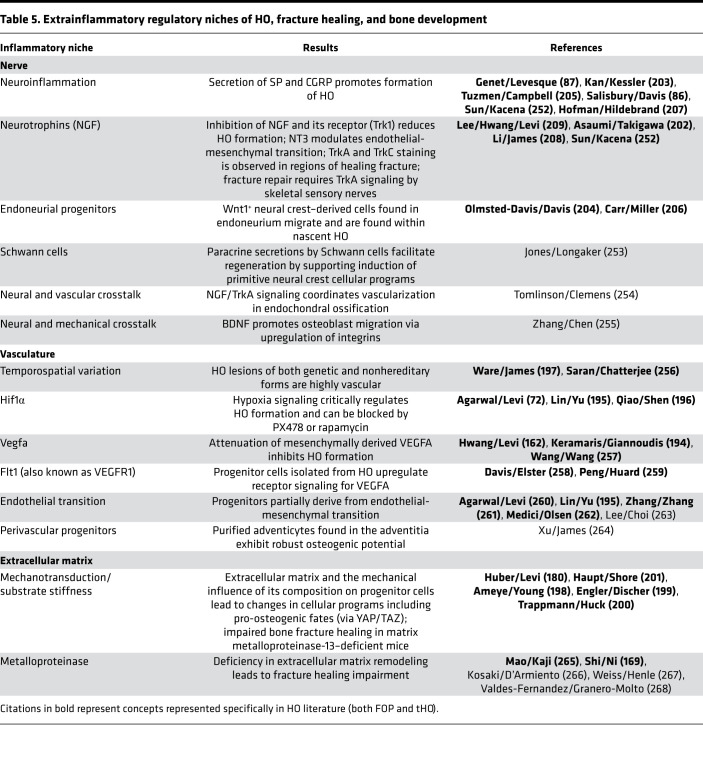
Extrainflammatory regulatory niches of HO, fracture healing, and bone development

**Table 4:**
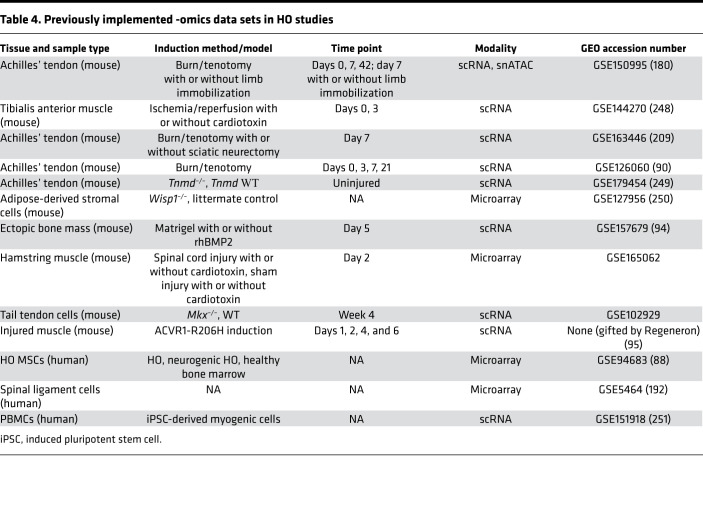
Previously implemented -omics data sets in HO studies

**Table 3:**
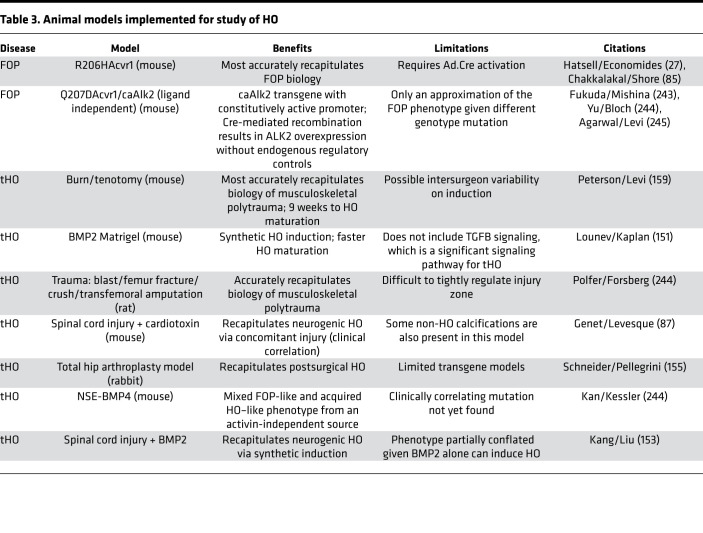
Animal models implemented for study of HO

**Table 1:**
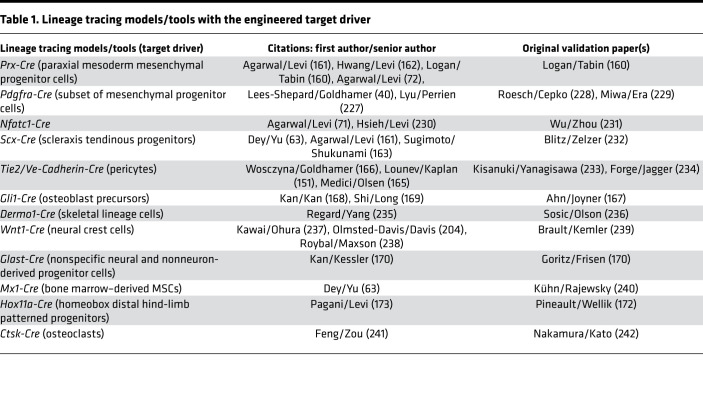
Lineage tracing models/tools with the engineered target driver

**Table 2:**
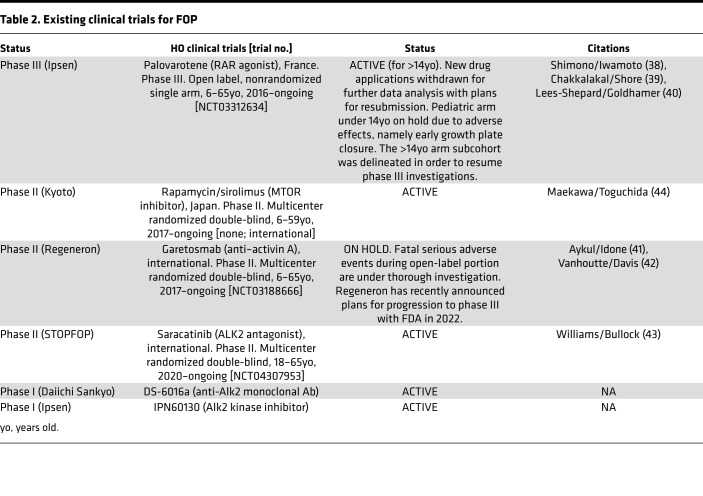
Existing clinical trials for FOP

## Version 1 07/22/2022

Electronic publication
